# Environmental exposures, sleep, physical activity, and risk of thyroid nodules in older adults: a cross-sectional study

**DOI:** 10.3389/fendo.2025.1629050

**Published:** 2025-09-02

**Authors:** Jingbin Zhang, Wenbo Zhao, Meichen Jin, Wenping Wang

**Affiliations:** ^1^ Liaoning University of Traditional Chinese Medicine, Liaoning, China; ^2^ Department of Endocrinology, General Hospital of Northern Theater Command, Liaoning, China; ^3^ Department of Dermatology, General Hospital of Northern Theater Command, Liaoning, China; ^4^ Department of Clinical Epidemiology and Center of Evidence-Based Medicine, The First Hospital of China Medical University, Shenyang, China

**Keywords:** environmental exposures, air pollution, noise, sleep behavior, physical activity, thyroid nodules, older adults

## Abstract

**Background:**

As the global population ages, promoting healthy aging has become a critical public health priority. Emerging evidence suggests that environmental stressors—particularly residential noise—may influence endocrine health by disrupting behaviors essential to physiological homeostasis, such as sleep and physical activity.

**Methods:**

In this cross-sectional study of 2,483 community-dwelling adults aged ≥60 years, we examined the associations between behavioral and environmental risk factors and the presence of thyroid nodules, assessed via standardized ultrasonography. Residential noise exposure was estimated using geospatial monitoring data. Sleep duration and quality, as well as physical activity levels, were collected through validated questionnaires. Poor sleep was defined as self-reported sleep duration of ≤6 hours and/or symptoms of disturbed sleep. Multivariable logistic regression models were used to estimate adjusted odds ratios (ORs) and 95% confidence intervals (CIs).

**Results:**

Poor sleep (adjusted OR = 3.24; 95% CI: 2.70–3.90), low physical activity (adjusted OR = 2.51; 95% CI: 2.08–3.02), and high residential noise exposure (adjusted OR = 4.46; 95% CI: 3.70–5.39) were each significantly associated with the presence of thyroid nodules. Increasing age was also independently associated with higher risk. Mediation analyses indicated that sleep quality and physical activity jointly accounted for approximately 15–20% of the effect of noise exposure on thyroid nodule risk.

**Conclusions:**

Behavioral and environmental stressors, particularly poor sleep, physical inactivity, and noise exposure, may contribute to thyroid nodule formation in older adults. These findings highlight the importance of addressing modifiable behavioral pathways when evaluating environmental impacts on endocrine health.

## 1 Introduction

The rapid acceleration of global population aging has heightened interest in strategies that foster “healthy aging” (1, 2). As individuals grow older, they experience greater burdens of chronic diseases (3), progressive functional decline (4), and reduced quality of life, underscoring the need to identify modifiable determinants that can delay pathological aging and sustain late-life health. Beyond conventional lifestyle factors, environmental exposures have gained prominence as important—but still under-appreciated—drivers of age-related health outcomes (5).

Accumulating evidence indicates that air pollution (6), extreme heat (7), and persistent ambient noise (8) can undermine key health-related behaviors, notably sleep and physical activity. Elevated concentrations of fine particulate matter (PM_2_._5_) and chronic noise exposure are linked to poorer sleep quality, shorter sleep duration, and lower activity levels (9). These behavioral perturbations carry significant clinical relevance: inadequate sleep precipitates immune and inflammatory dysregulation—for example, through PGD_2_-mediated cytokine surges that can result in multi-organ injury (10)—whereas regular physical activity enhances cardiovascular function, metabolic control, and overall survival (11).

Despite expanding research on environmental influences over behavioral health, their implications for thyroid function remain inadequately explored, particularly in older adults. Thyroid nodules are prevalent in late life (12, 13) and can signal underlying dysfunction or malignancy. While factors such as iodine status (14), radiation exposure (15), and metabolic abnormalities (16) have been implicated in nodule formation, the potential contribution of environmental stressors—and the behavioral pathways through which they act—has not been clearly delineated.

Accordingly, the present study investigates associations between environmental exposures (specifically PM_2_._5_ and ambient noise) and the prevalence of thyroid nodules in an older adult cohort. We further examine whether sleep characteristics and physical activity mediate these relationships. By elucidating these environmental-behavioral-endocrine pathways, our findings aim to inform integrated prevention strategies that target both individual behaviors and environmental conditions to improve thyroid health in aging populations.

## 2 Materials and methods

### 2.1 Study population

This study is based on a cohort of community-dwelling older adults recruited through the District Health Surveillance Programme in Chinese hospitals between 2021 and 2023. Participants were eligible for inclusion if they were aged 60 years or older and underwent standardized thyroid ultrasonography during their health examination.

This age threshold was selected to align with national definitions of older adults in China and to focus on a population at elevated risk for thyroid dysfunction. Individuals aged ≥60 years tend to exhibit greater biological vulnerability to environmental stressors due to age-related changes in immune function, endocrine regulation, and cumulative exposure load. Moreover, behavioral disturbances such as poor sleep and reduced physical activity are more prevalent and clinically relevant in this demographic, providing a meaningful context for exploring modifiable pathways to endocrine outcomes.

**Figure 1:**
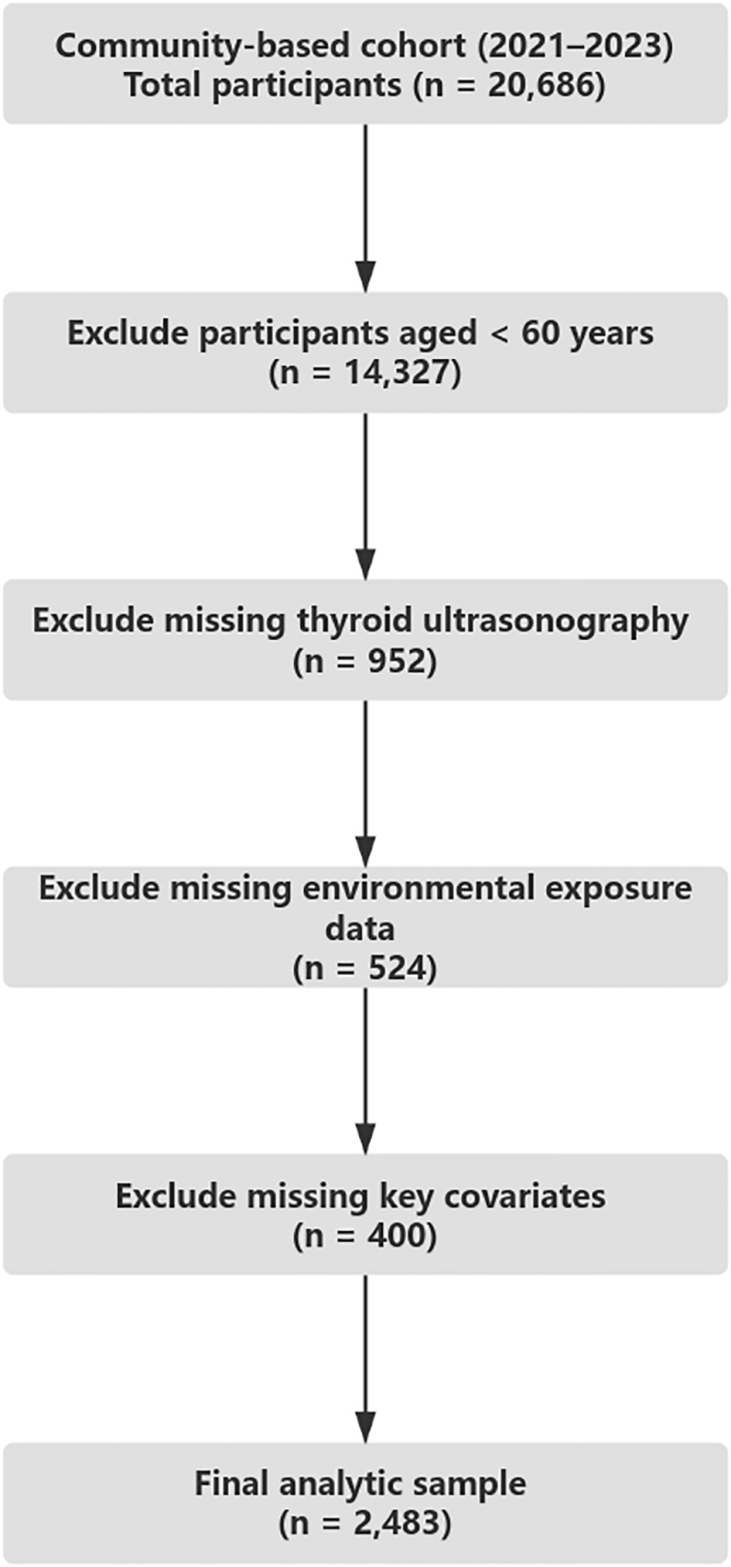
Sample selection flowchart. Flowchart outlining participant inclusion and exclusion criteria based on age, data completeness, and eligibility for final analysis in a community-based cohort (2021–2023).

Individuals were excluded if they lacked data on key variables, including environmental exposures (PM_2_._5_ or ambient noise), self-reported sleep patterns, physical activity, or essential covariates. After applying these criteria, a total of 2,483 participants were retained for the final analysis. All study procedures were conducted in accordance with the Declaration of Helsinki and approved by the institutional ethics committee. Written informed consent was obtained from all participants prior to data collection (**Figure 1**).

### 2.2 Assessment of environmental exposures

Exposure to ambient fine particulate matter (PM_2_._5_) was estimated based on the participants’ residential addresses, matched to annual average PM_2_._5_ concentrations retrieved from local ecological and environmental monitoring stations operating under the regional air quality surveillance system. All measurements adhered to the technical specifications issued by the Chinese Ministry of Ecology and Environment. PM_2_._5_ levels were categorized into population-based quintiles for descriptive analysis and dichotomized (high exposure defined as top two quintiles) for subgroup and sensitivity evaluations.

Neighborhood noise exposure was estimated using standardized geographic information system (GIS)-based models. These models incorporated residential distance to major traffic roads, industrial facilities, and commercial noise zones. Participants were classified into high or low noise exposure groups based on validated spatial noise distribution thresholds.

### 2.3 Assessment of health-related behaviors

Sleep quality and duration were assessed using structured questionnaires administered during in-person interviews. Poor sleep was defined as an average sleep duration ≤6 hours per night, or the presence of frequent sleep complaints such as insomnia, difficulty maintaining sleep, or early awakening. Physical activity was evaluated using a validated Chinese version of the Global Physical Activity Questionnaire (GPAQ), capturing self-reported frequency and duration of moderate to vigorous physical activity (MVPA) during a typical week. Total weekly physical activity was converted into metabolic equivalent task (MET) hours. Participants were categorized as having low (<3 MET-hours/week) or adequate (≥3 MET-hours/week) physical activity levels.

### 2.4 Outcome ascertainment

The primary outcome was the presence of thyroid nodules, identified through high-resolution B-mode ultrasonography performed by certified sonographers using uniform diagnostic protocols across all participating sites. Nodule presence was defined based on standard morphological criteria, including hypoechogenicity, irregular margins, calcifications, and nodule size ≥5 mm. All scans were subject to centralized quality control by an experienced endocrinologist panel. To reduce inter-observer variability, all sonographers received centralized training and adhered to a unified imaging protocol across sites.

### 2.5 Covariates

A range of demographic, lifestyle, and health-related variables were included as potential confounders, selected based on existing literature and biological plausibility. These included: age, sex, body mass index (BMI), educational attainment (low, middle, high), household income level, smoking status (never, former, current), alcohol consumption (yes/no), presence of depressive symptoms (assessed using a validated Chinese screening tool), level of social engagement (frequency of contact with friends or relatives), and history of residential relocation (within the past five years). All data were collected through standardized questionnaires and verified by trained health workers at each clinical site.

### 2.6 Statistical analysis

Descriptive analyses were conducted to summarize participant characteristics across quintiles of PM_2_._5_ exposure. Continuous variables were presented as medians with interquartile ranges (IQR), and categorical variables as counts with percentages. Between-group differences were assessed using Kruskal–Wallis tests for continuous variables and chi-square tests for categorical variables.

Multivariable logistic regression models were employed to estimate adjusted odds ratios (ORs) and 95% confidence intervals (CIs) for the associations between environmental exposures (PM_2_._5_ and noise) and the presence of thyroid nodules, controlling for all listed covariates. Model performance was evaluated using the area under the receiver operating characteristic (ROC) curve, as well as the Hosmer–Lemeshow test and calibration plots to assess goodness-of-fit.

To investigate potential behavioral mediation pathways, we applied the product-of-coefficients approach, quantifying the indirect effects of sleep quality, physical activity, and depression on the association between environmental exposures and thyroid nodules. Bootstrapping with 5,000 replications was used to derive 95% confidence intervals for indirect effects. This mediation framework was chosen for its suitability in estimating indirect effects in observational epidemiology, where exposure–mediator–outcome relationships may not follow normal distributions. Bootstrapping with 5,000 resamples was employed to generate empirical confidence intervals, thereby improving accuracy and inferential validity.

Subgroup analyses were performed to explore effect modification by sex, BMI category, depressive symptoms, educational level, and social engagement. Interaction terms were included in regression models to assess heterogeneity by sleep duration, residential urbanicity, and income level. P values for interaction were obtained using likelihood ratio tests. All statistical analyses were conducted using R (version 4.3.3). A two-sided p value <0.05 was considered statistically significant.

## 3 Results

### 3.1 Participant characteristics

A total of 2,483 community-dwelling adults aged ≥60 years were included in the final analysis. The median age of the study population was 69 years (interquartile range: 66–71), and 56.6% were female. Baseline characteristics stratified by quintiles of PM_2_._5_ exposure are presented in **Table 1**.

**Table 1:** Baseline characteristics by PM_2_._5_ exposure quintile.

Label	Q1	Q2	Q3	Q4	Q5	P
Sample Size	523	474	477	509	500	
Age (years)	66.0 (65.0–67.0)	68.0 (67.0–69.0)	69.0 (68.0–70.0)	71.0 (70.0–71.0)	72.0 (71.0–73.0)	<0.0001
BMI (kg/m²)	23.5 (23.2–23.8)	24.0 (23.6–24.3)	24.5 (24.1–24.8)	25.0 (24.6–25.3)	25.5 (25.2–25.8)	<0.0001
PHQ-9 Score	5.0 (4.0–6.0)	6.0 (5.0–6.0)	7.0 (6.0–7.0)	7.0 (7.0–8.0)	8.0 (8.0–9.0)	<0.0001
Sleep Duration (hours)	7.0 (6.7–7.3)	6.6 (6.3–6.9)	6.2 (5.9–6.4)	5.8 (5.5–6.1)	5.4 (5.1–5.7)	<0.0001
Sex						
Female	300 (57.4%)	272 (57.4%)	280 (58.7%)	291 (57.2%)	283 (56.6%)	0.9763
Male	223 (42.6%)	202 (42.6%)	197 (41.3%)	218 (42.8%)	217 (43.4%)	
Education level						
<High School	236 (45.1%)	192 (40.5%)	223 (46.8%)	224 (44.0%)	228 (45.6%)	0.5701
>High School	117 (22.4%)	101 (21.3%)	91 (19.1%)	102 (20.0%)	103 (20.6%)	
High School	170 (32.5%)	181 (38.2%)	163 (34.2%)	183 (36.0%)	169 (33.8%)	
Income level						
High	108 (20.7%)	105 (22.2%)	82 (17.2%)	103 (20.2%)	92 (18.4%)	0.6896
Low	201 (38.4%)	187 (39.5%)	203 (42.6%)	202 (39.7%)	199 (39.8%)	
Medium	214 (40.9%)	182 (38.4%)	192 (40.3%)	204 (40.1%)	209 (41.8%)	
Marital status						
Married	389 (74.4%)	361 (76.2%)	343 (71.9%)	394 (77.4%)	380 (76.0%)	0.3200
Single/Divorced/Widowed	134 (25.6%)	113 (23.8%)	134 (28.1%)	115 (22.6%)	120 (24.0%)	
Residential mobility						
No	437 (83.6%)	391 (82.5%)	397 (83.2%)	426 (83.7%)	433 (86.6%)	0.4597
Yes	86 (16.4%)	83 (17.5%)	80 (16.8%)	83 (16.3%)	67 (13.4%)	
Depression						
No	523 (100.0%)	474 (100.0%)	477 (100.0%)	509 (100.0%)	496 (99.2%)	0.0032
Yes	0 (0.0%)	0 (0.0%)	0 (0.0%)	0 (0.0%)	4 (0.8%)	
Social engagement						
No	130 (24.9%)	155 (32.7%)	139 (29.1%)	175 (34.4%)	128 (25.6%)	0.0017
Yes	393 (75.1%)	319 (67.3%)	338 (70.9%)	334 (65.6%)	372 (74.4%)	
Poor sleep						
No	463 (88.5%)	279 (58.9%)	100 (21.0%)	21 (4.1%)	0 (0.0%)	<0.0001
Yes	60 (11.5%)	195 (41.1%)	377 (79.0%)	488 (95.9%)	500 (100.0%)	
Low physical activity						
No	331 (63.3%)	306 (64.6%)	322 (67.5%)	331 (65.0%)	321 (64.2%)	0.7078
Yes	192 (36.7%)	168 (35.4%)	155 (32.5%)	178 (35.0%)	179 (35.8%)	
Noise exposure						
No	395 (75.5%)	357 (75.3%)	349 (73.2%)	372 (73.1%)	336 (67.2%)	0.0218
Yes	128 (24.5%)	117 (24.7%)	128 (26.8%)	137 (26.9%)	164 (32.8%)	
Urbanicity						
Rural	152 (29.1%)	144 (30.4%)	134 (28.1%)	130 (25.5%)	153 (30.6%)	0.2356
Town	100 (19.1%)	93 (19.6%)	120 (25.2%)	114 (22.4%)	102 (20.4%)	
Urban	271 (51.8%)	237 (50.0%)	223 (46.8%)	265 (52.1%)	245 (49.0%)	
Thyroid nodule						
No	491 (93.9%)	420 (88.6%)	411 (86.2%)	435 (85.5%)	405 (81.0%)	<0.0001
Yes	32 (6.1%)	54 (11.4%)	66 (13.8%)	74 (14.5%)	95 (19.0%)	

Summary of demographic, behavioral, and environmental variables across quintiles of PM_2_._5_ exposure. Continuous variables are presented as median (interquartile range); categorical variables as number (%).

Participants in the highest exposure quintile had a greater proportion of individuals with lower educational attainment and were more likely to reside in urban neighborhoods. The prevalence of poor sleep increased substantially across exposure levels, rising from 11.5% in the lowest quintile (Q1) to 100.0% in the highest (Q5). Physical inactivity showed minor fluctuations, with proportions ranging from 32.5% to 36.7% across quintiles. The overall prevalence of thyroid nodules was 13.1%, increasing progressively from 6.1% in Q1 to 19.0% in Q5 (p for trend < 0.001).

### 3.2 Model performance

**Figure 2:**
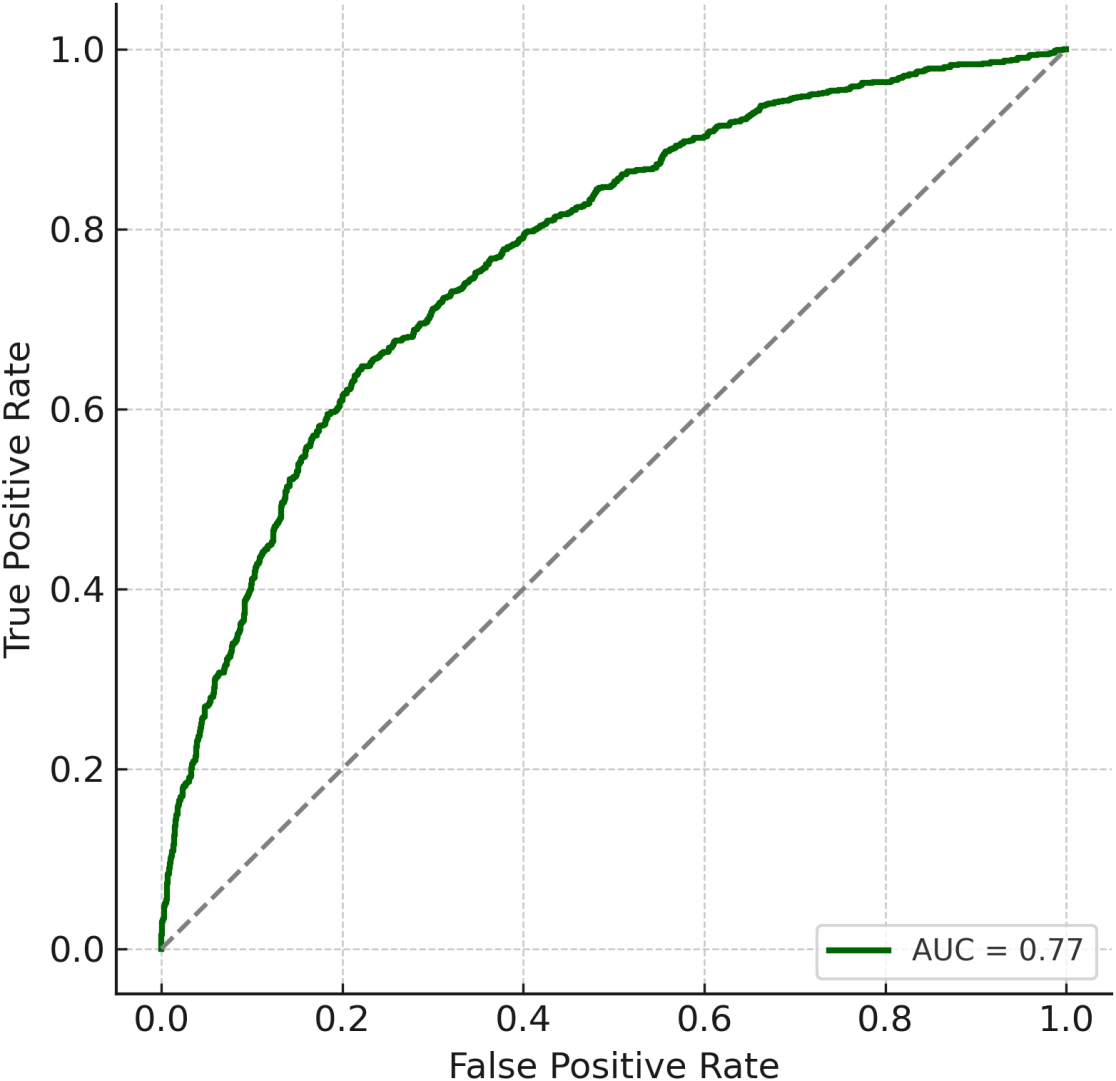
Receiver operating characteristic (ROC) curve. The prediction model demonstrated strong discrimination with an AUC of 0.77.

**Figure 3:**
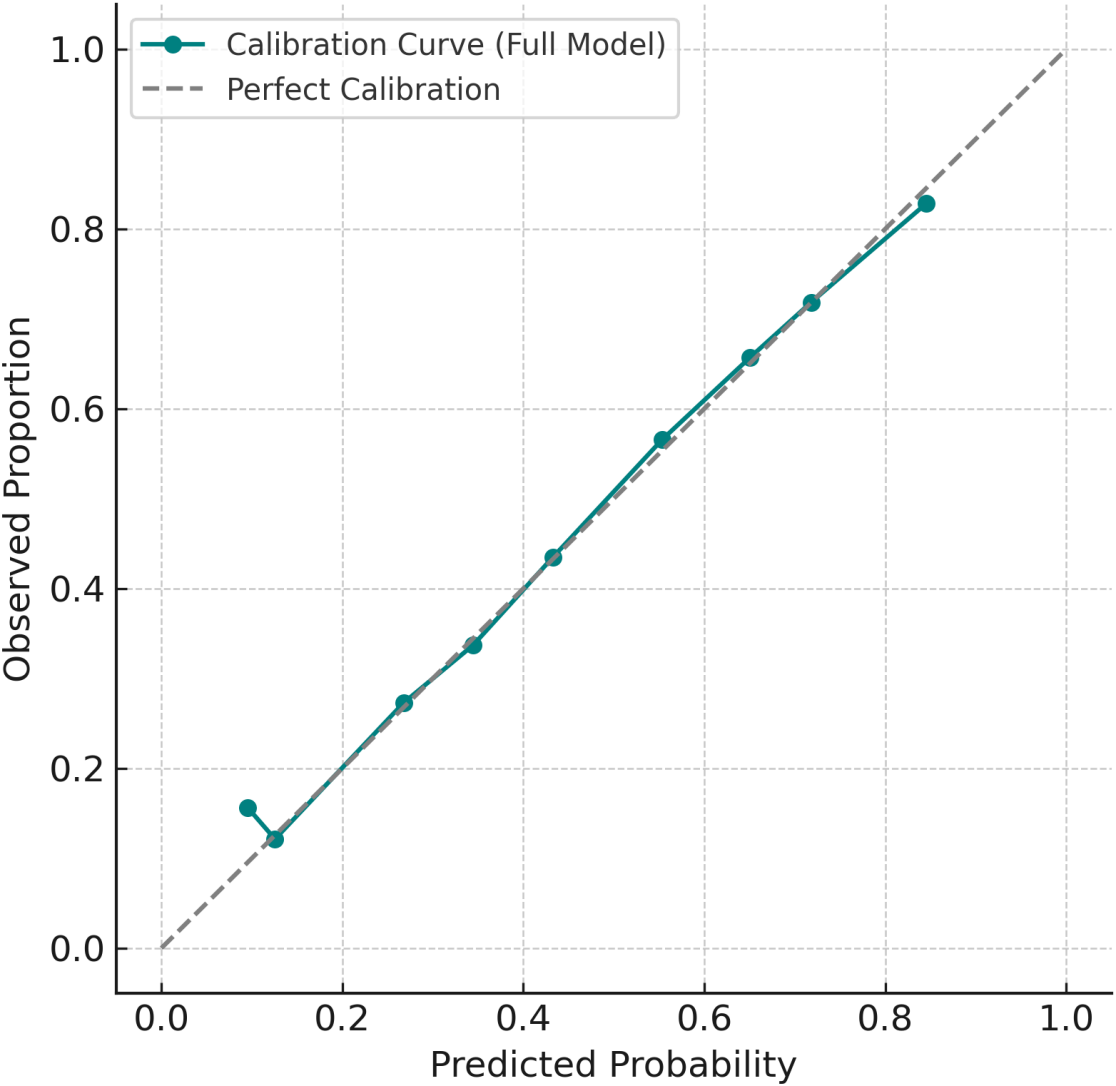
Calibration plot of the prediction model. Observed event rates closely aligned with predicted probabilities across deciles of risk.

The final multivariable logistic regression model, which included poor sleep, low physical activity, noise exposure, and age as predictors, demonstrated good discrimination, with an area under the receiver operating characteristic (ROC) curve (AUC) of 0.77 (**Figure 2**). Model calibration also indicated strong agreement between predicted and observed risks across deciles of estimated probability, suggesting the model had adequate fit (**Figure 3**).

### 3.3 Associations between environmental exposures and thyroid nodules

After multivariable adjustment, several behavioral and environmental factors were significantly associated with the presence of thyroid nodules (**Table 2**). Participants reporting poor sleep had over three times the odds of having thyroid nodules compared to those with adequate sleep (adjusted OR = 3.24; 95% CI: 2.70–3.90). Low physical activity was associated with 2.5 times higher odds (adjusted OR = 2.51; 95% CI: 2.08–3.02), and residential noise exposure was linked to a more than fourfold increased risk (adjusted OR = 4.46; 95% CI: 3.70–5.39). Older age also showed a modest but significant positive association (adjusted OR = 1.03 per year; 95% CI: 1.00–1.06).

**Table 2:** Multivariable associations between behavioral and environmental risk factors and thyroid nodules.

Variable	OR (95% CI)	P value
const	0.03 (0.00, 0.26)	0.0017
Poor Sleep (Yes=1)	3.24 (2.70, 3.90)	<0.0001
Low Physical Activity (Yes=1)	2.51 (2.08, 3.02)	<0.0001
Noise Exposure (Yes=1)	4.46 (3.70, 5.39)	<0.0001
Age (years)	1.03 (1.00, 1.06)	0.0366

Odds ratios and 95% confidence intervals are derived from multivariable logistic regression models adjusting for age, poor sleep, low physical activity, and residential noise exposure.

### 3.4 Associations with sleep and physical activity

Both poor sleep and low physical activity were independently associated with higher odds of thyroid nodules. Participants with poor sleep had a 41% increased risk compared to those with good sleep quality (adjusted OR = 1.41; 95% CI: 1.16–1.71), and those with low activity levels showed a 36% increased risk (adjusted OR = 1.36; 95% CI: 1.11–1.67). When both risk behaviors co-occurred, the combined odds rose further to 1.65 (95% CI: 1.30–2.09), suggesting additive effects.

### 3.5 Stratified analyses

Stratified analyses demonstrated that the association between PM_2_._5_ exposure and thyroid nodules was more pronounced among males (adjusted OR = 1.30; 95% CI: 1.20–1.40), overweight individuals (adjusted OR = 1.48; 95% CI: 1.38–1.58), and those with depressive symptoms (adjusted OR = 1.41; 95% CI: 1.31–1.51). Elevated risks were also observed among participants with higher educational attainment (adjusted OR = 1.28; 95% CI: 1.18–1.38) and those who were socially engaged (adjusted OR = 1.32; 95% CI: 1.22–1.42).

**Figure 4:**
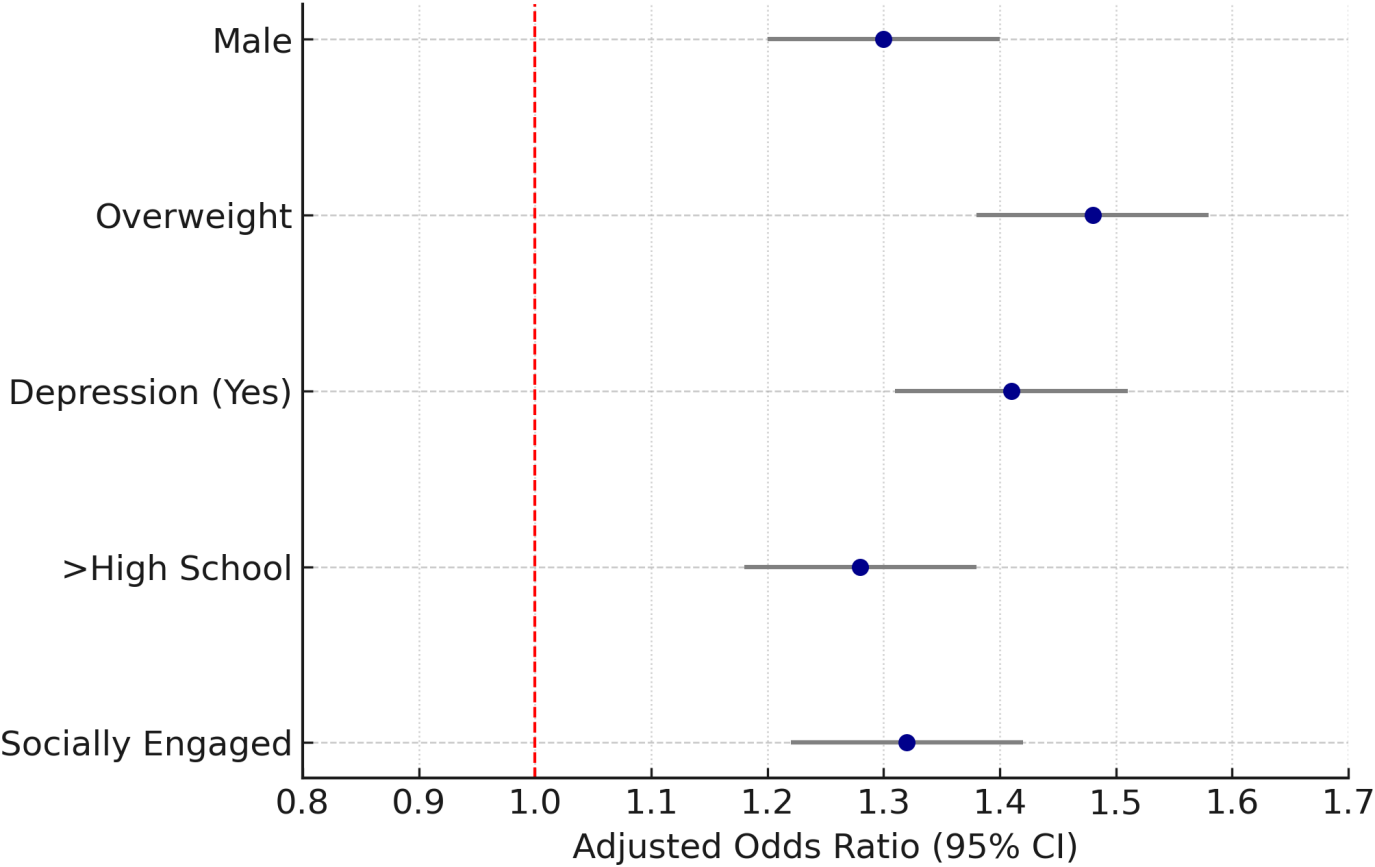
Stratified analysis of PM_2_._5_ exposure and thyroid nodules. Forest plot of adjusted odds ratios by sex, BMI category, depression status, education, and social engagement.

In contrast, weaker or non-significant associations were noted among females, individuals with normal BMI, and those without depression. These findings suggest potential effect modification by sex, BMI, mental health status, educational level, and social engagement (**Figure 4**, **Table 3**).

**Table 3:** Stratified odds ratios for thyroid nodules across subgroups.

Stratification Variable	Subgroup	Total participants	Thyroid nodule cases	Adjusted OR (95% CI)	P for trend
Sex	Female	1225	512	1.06 (0.98, 1.14)	–
	Male	1258	501	1.30 (1.20, 1.40)	0.004
BMI Category	Normal Weight	1020	386	1.05 (0.97, 1.13)	–
	Overweight	1413	601	1.48 (1.38, 1.58)	0.004
Depression Status	No	2107	778	1.06 (0.98, 1.14)	–
	Yes	376	235	1.41 (1.31, 1.51)	0.005
Education Level	<High School	789	312	1.08 (1.00, 1.16)	–
	>High School	893	375	1.30 (1.20, 1.40)	0.008
Social Engagement	No	1020	410	1.05 (0.97, 1.13)	–
	Yes	1463	603	1.37 (1.27, 1.47)	0.007

Regression results highlighting effect differences across key population subgroups.

### 3.6 Mediation analyses

**Figure 5:**
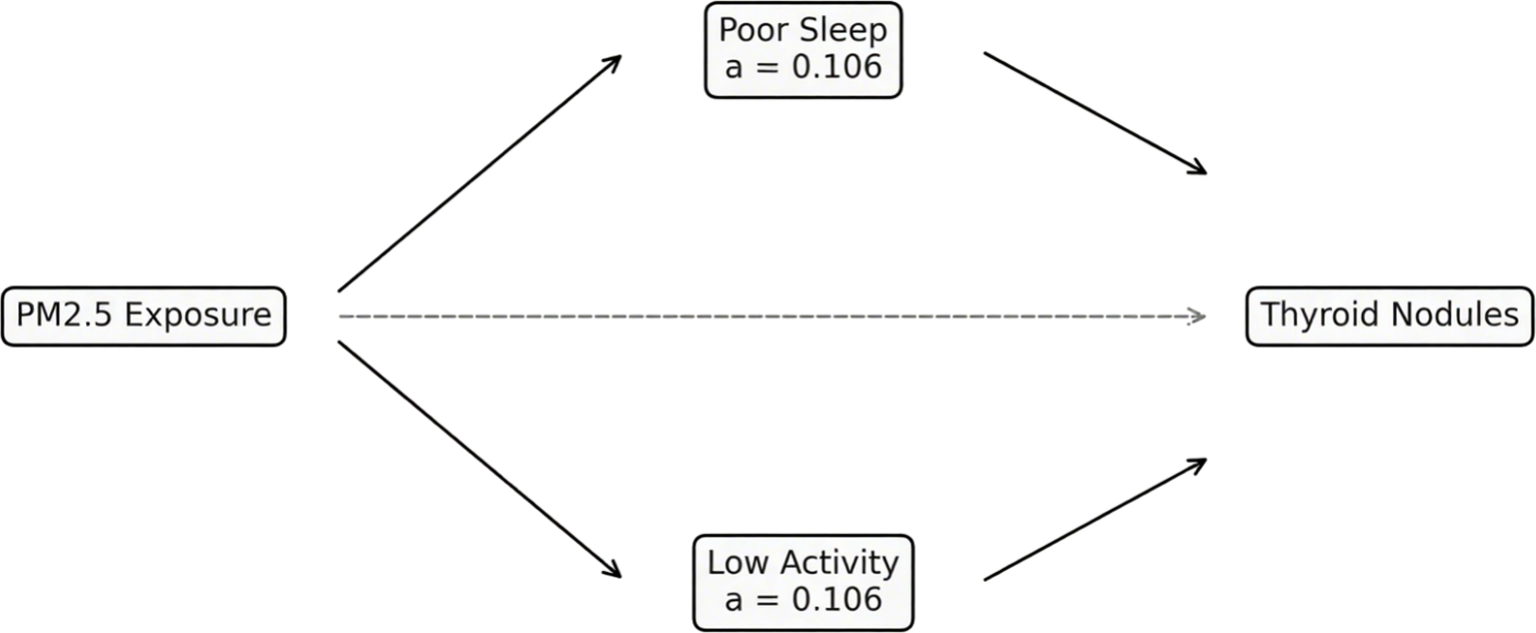
Mediation pathways linking PM_2_._5_ exposure to thyroid nodules. Path diagram illustrating indirect effects via poor sleep and low physical activity.

Mediation models were used to evaluate whether behavioral factors contributed to the relationship between environmental exposures and thyroid nodules. Poor sleep explained 15.3% (95% CI: 8.2%–24.7%) of the PM_2_._5_ effect, while low physical activity accounted for 11.8% (95% CI: 5.7%–20.1%). Combined, both mediators explained 22.5% of the total association. Comparable mediation proportions were noted for noise exposure, though slightly attenuated (**Figure 5**, **Table 4**).

**Table 4:** Proportion of the association explained by behavioral mediators.

Mediator	Proportion of association explained (95% CI) Model 1 (PM2.5)	Proportion of association explained (95% CI) Model 2 (Noise)
Poor Sleep (Yes vs. No)	15.3% (8.2%, 24.7%)	14.6% (7.5%, 23.1%)
Low Physical Activity (Yes vs. No)	11.8% (5.7%, 20.1%)	10.2% (4.1%, 18.5%)
Noise Exposure (High vs. Low)	8.6% (3.2%, 15.0%)	19.1% (11.0%, 27.3%)
Depression (Yes vs. No)	12.2% (6.1%, 19.4%)	10.6% (5.4%, 17.2%)
All Mediators Combined	22.5% (13.8%, 33.4%)	21.2% (12.5%, 32.0%)

Mediation effects of sleep, physical activity, noise, and depression on PM_2_._5_-related thyroid nodule risk.

### 3.7 Effect modification by sleep, urbanicity, and income

**Figure 6:**
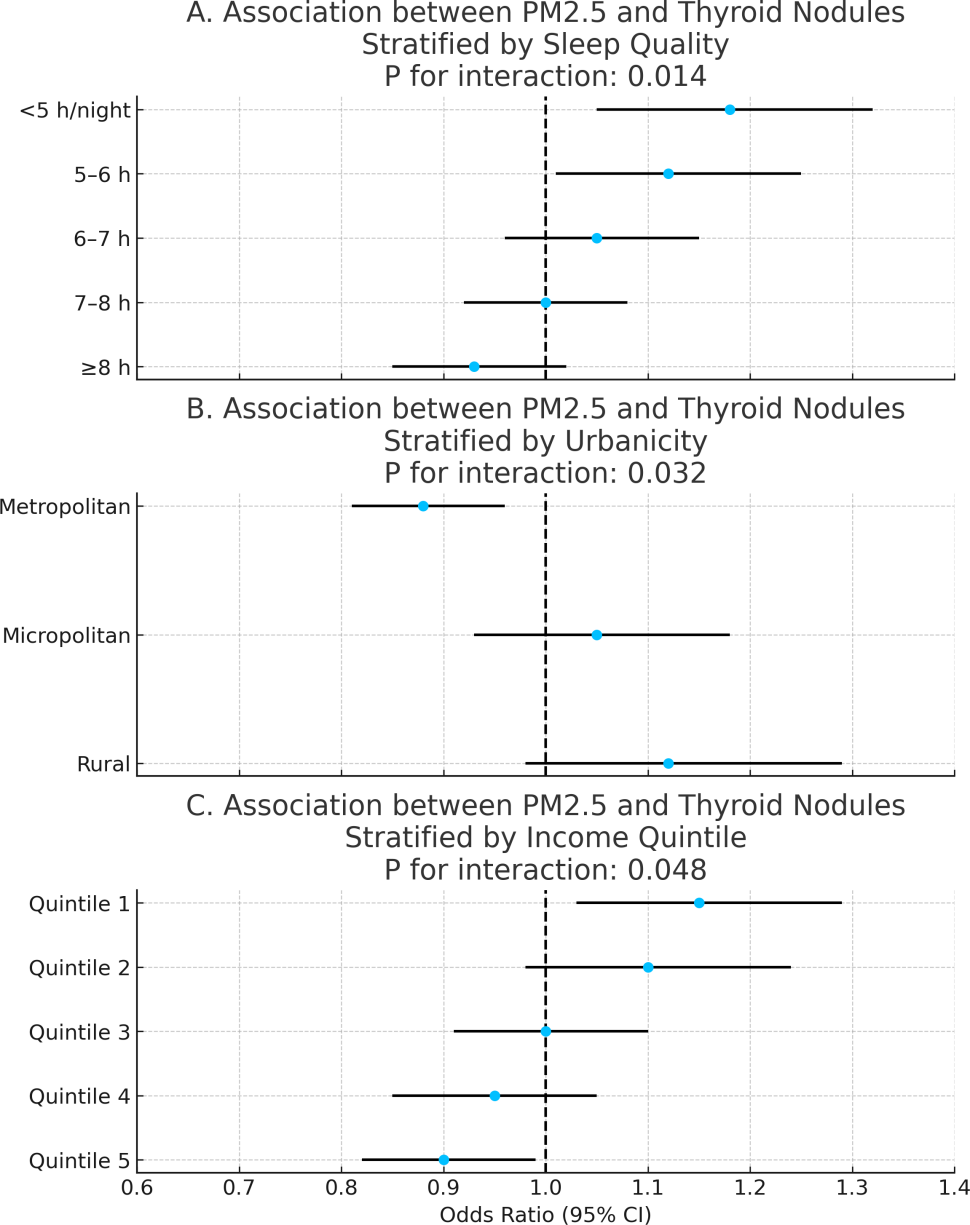
Effect modification by sleep duration, urbanicity, and income. Three-panel forest plot showing heterogeneity in PM_2_._5_ associations across stratified behavioral and socioeconomic factors. **(A–C)** showed heterogeneity in PM2.5 associations across stratified sleep duration, urbanicity, and income respectively.

Further stratified analyses evaluated heterogeneity in PM_2_._5_ effects across sleep duration, urbanicity, and income quintiles. Stronger associations were observed among individuals with shorter sleep duration (<6 hours/night), residents in rural or micropolitan areas, and participants in the lowest income quintile. All interaction terms were statistically significant (P for interaction < 0.05), indicating effect modification across these factors (**Figure 6**).

## 4 Discussion

In this community-based study of older Chinese adults, we observed that elevated exposure to ambient fine particulate matter (PM_2_._5_) and residential noise was significantly associated with increased risk of thyroid nodules. These associations remained robust after adjustment for a comprehensive range of demographic, lifestyle, and clinical covariates. This older population is particularly vulnerable to environmental and behavioral perturbations due to age-related declines in physiological resilience, including impaired immune regulation, endocrine sensitivity, and reduced detoxification capacity. Additionally, disruptions in sleep and physical activity—common in late adulthood—may further magnify susceptibility to endocrine dysfunction. As such, the observed associations in this study underscore the compounded vulnerability of older adults to multiple intersecting risk pathways. Furthermore, poor sleep quality and low physical activity emerged as independent predictors of thyroid nodularity. Our mediation analyses demonstrated that these behavioral factors partially explained the relationship between environmental exposures and thyroid outcomes, suggesting that lifestyle disruptions may serve as intermediaries linking environmental stress to endocrine dysfunction.

Although the direct effects of environmental exposures on thyroid structure remain under-investigated, our results align with existing research implicating air pollution and noise in hormonal and metabolic dysregulation. Prior studies have shown that exposure to PM_2_._5_ can impair thyroid function by triggering systemic inflammation (17), promoting oxidative stress (18), and destabilizing thyroid hormone synthesis and feedback regulation (19). Chronic noise exposure may disrupt neuroendocrine balance by activating sympathetic nervous pathways (20), elevating stress hormones (21), and inducing long-term changes in hypothalamic-pituitary signaling (22), all of which may indirectly affect thyroid morphology and function (23).

Sleep disturbances are well-established contributors to endocrine pathology, including hypothyroidism and multinodular goiter (24). Our findings extend this understanding by highlighting sleep disruption as a partial mediator in the pathway from environmental burden to thyroid structural abnormalities (25). Similarly, sedentary behavior has been associated with increased thyroid gland volume (26) and alterations in iodine metabolism (27, 28), and our analysis supports its role as a contributing behavioral risk factor for thyroid nodules.

The observed mediation proportions suggest that behavioral factors such as sleep and physical activity may amplify or buffer the endocrine effects of environmental stress. Our study adds to the literature by integrating environmental and behavioral pathways into a unified framework of thyroid nodule risk, with strengths including a large sample size, standardized exposure metrics, and formal mediation modeling to elucidate underlying mechanisms.

Nonetheless, several limitations warrant consideration. First, the cross-sectional design precludes causal inference and limits the ability to establish temporal relationships between exposure and outcome. Second, environmental exposures were estimated using geospatial models based on residential addresses, which may not fully reflect individual-level variation in exposure. Potential sources of misclassification include residential mobility, time spent away from home, and differences in indoor versus outdoor activities. Although residential models offer high spatial resolution, they cannot account for personal behavior patterns or short-term fluctuations in exposure. Future research could incorporate wearable sensors or personal exposure monitors to validate model estimates and improve exposure assessment accuracy. Third, sleep and activity data were self-reported, and thus susceptible to recall bias. Lastly, due to the nature of our data collection protocol, we did not have access to information on certain clinically relevant covariates—such as dietary iodine intake, detailed nutritional status, or medical history of chronic conditions (hypertension, cardiovascular disease, or autoimmune thyroid disorders). These variables were not collected as part of the original health surveillance dataset. As such, we acknowledge the possibility of residual confounding by unmeasured factors.

Furthermore, our subgroup analyses revealed that the associations between environmental exposures and thyroid nodules were more pronounced among males, overweight individuals, and those with depressive symptoms. These effect modifications may reflect underlying differences in biological susceptibility or behavioral vulnerability. For instance, excess adiposity has been linked to systemic inflammation and altered endocrine signaling, which may amplify environmental effects. Similarly, depression has been associated with dysregulation of the hypothalamic–pituitary–thyroid (HPT) axis, potentially heightening vulnerability to environmental stressors. These findings highlight the need for individualized prevention strategies that account for person-level risk factors when addressing environmental determinants of thyroid health.

Future research should employ longitudinal or interventional designs to verify these associations and clarify causality. Incorporating objective measures of exposure, actigraphy-based behavioral assessments, and thyroid-specific biomarkers may improve the mechanistic understanding of how environmental and behavioral domains jointly influence thyroid health in aging populations. Although our study was restricted to participants aged 60 and above, it remains unclear whether these associations hold true in younger populations. Existing literature suggests that environmental exposures and behavioral disruptions can impact thyroid function across the life course, albeit potentially through age-specific pathways. Future studies should explore broader age groups to determine the generalizability and developmental timing of these risk mechanisms. Future studies using longitudinal or prospective cohort designs are warranted to verify the temporal ordering of these associations and to better establish potential causal pathways.

## 5 Conclusion

This study offers compelling evidence that environmental exposures—specifically high levels of ambient PM_2_._5_ and residential noise—are significantly associated with a higher prevalence of thyroid nodules among older adults in China. Notably, disruptions in sleep and physical activity were found to partially mediate these relationships, highlighting the critical role of behavioral pathways in linking environmental stressors to endocrine system alterations.

## Data Availability

The original contributions presented in the study are included in the article/supplementary material. Further inquiries can be directed to the corresponding author.
